# Emerging Biomarkers for Diagnosis, Prevention and Treatment of Brain Metastases—From Biology to Clinical Utility

**DOI:** 10.3390/diseases10010011

**Published:** 2022-02-03

**Authors:** Priyakshi Kalita-de Croft, Vaibhavi Joshi, Jodi M. Saunus, Sunil R. Lakhani

**Affiliations:** 1 UQ Centre for Clinical Research, The University of Queensland Faculty of Medicine, Herston, QLD 4029, Australia; vaibhavi.joshi@uq.edu.au (V.J.); j.saunus@uq.edu.au (J.M.S.); 2 Pathology Queensland, The Royal Brisbane and Women’s Hospital Herston, Herston, QLD 4029, Australia

**Keywords:** liquid biopsy, brain metastasis, circulating tumour cells, extracellular vesicles, diagnostic, predictive, prognostic

## Abstract

Primary malignancies of the lung, skin (melanoma), and breast have higher propensity for metastatic spread to the brain. Advances in molecular tumour profiling have aided the development of targeted therapies, stereotactic radiotherapy, and immunotherapy, which have led to some improvement in patient outcomes; however, the overall prognosis remains poor. Continued research to identify new prognostic and predictive biomarkers is necessary to further impact patient outcomes, as this will enable better risk stratification at the point of primary cancer diagnosis, earlier detection of metastatic deposits (for example, through surveillance), and more effective systemic treatments. Brain metastases exhibit considerable inter- and intratumoural heterogeneity—apart from distinct histology, treatment history and other clinical factors, the metastatic brain tumour microenvironment is incredibly variable both in terms of subclonal diversity and cellular composition. This review discusses emerging biomarkers; specifically, the biological context and potential clinical utility of tumour tissue biomarkers, circulating tumour cells, extracellular vesicles, and circulating tumour DNA.

## 1. Introduction

Brain cancers can be primary, arising within different areas of the brain, or metastatic, arising from different organs of the body and spreading to the brain, also known as brain metastasis (BrM). This review focuses on discussing BrM-related biomarkers. These metastatic tumours are most frequent in lung cancers, followed by breast, melanoma, colon, kidney, and ovarian cancers, and 15% of cases with unknown primary origin [1]. Over the past 40 years, the quality of life and outcomes of patients with BrM have improved, owing to progress in neuroimaging, neurosurgery, and oncology. Magnetic resonance imaging is a standard procedure for diagnosis. BrMs usually appear as contrast-enhancing lesions, most frequently in the cerebral hemispheres (80% of cases), cerebellum (15%), and brainstem (<5%) [2]. Under a newly revised graded prognostic assessment [3], BrM patient survival rates have improved; however, they are still variable. For lung cancer, median overall survival is 15 months, 14–16 months for breast, 7–10 months for melanoma, 5–8 months for GI cancers, and 10–12 months for renal cell carcinoma [4]. This variability reflects the heterogenous patient population and highlights the complexities of clinical management.

## 2. Current Clinical Management Strategies for Brain Metastases

The management of BrM patients is usually coordinated in a multidisciplinary team setting, with treatment planning based on patient age, Karnofsky performance status (a general well-being scale), the primary origin of the cancer as some primaries have a higher risk of BrM, and the extent of extracranial disease control [5,6]. Corticosteroids and antiepileptic agents are routinely used for neurological symptom management. In terms of local treatment, neurosurgical tumour resection is considered depending on the location and the number of metastases within the brain (oligometastatic; limited metastatic burden or highly metastatic), with contemporary surgical interventions such as cortical mapping [7] and laser interstitial thermal therapy [8] used to minimize perioperative morbidity. Irradiating the whole brain remains a critical option when surgery is not appropriate, but stereotactic radiosurgery (SRS) is preferred for patients with up to or more than four BrM [9] and good performance status because it targets radiation very precisely to minimize treatment-induced neurocognitive side effects.

Various guidelines are available for treatment of brain metastasis from solid tumours, including the European Association of Neuro-Oncology (EANO)–European Society for Medical Oncology (ESMO) [10] and the National Comprehensive Cancer network (NCCN) [11]. Guidelines from the NCCN define BrM patients to be treated with surgery, stereotactic radiosurgery (SRS), whole-brain radiation therapy (WBRT), and systemic therapy [11]. However, new recommendations for patients presenting with extensive brain metastasis, usually with a known history of cancer, undergo either resection or biopsy to confirm CNS involvement, and then they may be subjected to WBRT, SRS, or systemic therapy and then follow-up with brain MRI every 2–3 months for the next 1–2 years. NCCN encourages that patients with limited metastatic lesions should undergo a prior consultation phase with a multidisciplinary team to optimise the best treatment plan. For instance, patients with disseminated systemic disease with poor response may undergo WBRT or SRS or perhaps consider palliative care. Conversely, patients with newly diagnosed or stable systemic disease may undergo SRS or WBRT. Nonetheless, then these patients will be followed up by brain MRI every 2–3 months for 1–2 years and every 4–6 months indefinitely. Furthermore, depending on either recurrence locally or in distant sites, they might be evaluated for further surgery, SRS, WBRT, and systemic therapy [11]. The systemic therapy regimen usually depends on the primary tumour, for instance, BrM patients with breast primaries are treated with breast cancer regimen on the basis of the breast cancer subtype, lung BrM patients are given lung cancer regimens, and so on. Overall, systemic therapy such as chemotherapeutics such as cisplatin, paclitaxel, and temozolomide have shown mixed results in clinical trials [12,13]; however, they are still used in practice if they can help stabilize extracranial metastases and they may have an impact in the brain in some patients. Any prescription of molecular-targeted therapy is based on primary histology, treatment history, and data on actionable alterations where available. For example, trastuzumab is used for HER2-amplified breast cancer BrM, tragisso (Osimertinib) for EGFR-mutant lung cancer BrM [14,15], and xalkori (Crizotinib), alecensa (Alectinib), alunbrig (Brigatinib), and zykadia (Ceritinib) against ALK-rearranged BrM [4,15,16,17,18,19,20,21]. However, molecular-targeted agents are generally also given with the goal of stabilising, rather than eliminating, metastatic deposits, as complete intracranial responses are rare. Factors that limit the efficacy of the existing treatments include intratumoural heterogeneity, insufficient vascular permeability due to the blood–brain barrier (BBB), elevated tumour interstitial fluid pressure, and other aspects of the tumour microenvironment (TME) [22,23,24,25].

**Figure 1:**
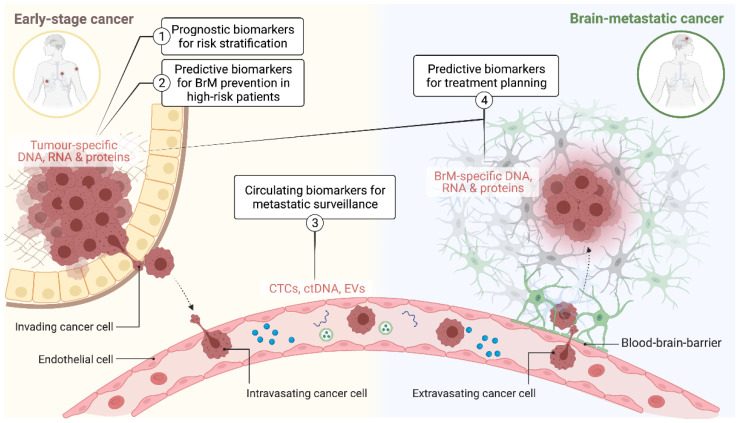
Schematic illustrating the application of predictive and prognostic biomarkers at different stages of brain metastasis pathogenesis. CTCs: circulating tumour cells; ctDNA: circulating tumour DNA; EVs: extracellular vesicles; BrM: brain metastasis.

For new experimental agents, increasing the number of phase II and III trials that specifically test intracranial efficacy as a secondary endpoint will be important if we are to increase the number of options available in the brain metastatic setting [26,27]. Developing companion biomarkers in parallel to molecular target discovery and drug development is an essential component of this (Figure 1). This includes diagnostic tools for more accurate risk stratification and earlier intervention for low-stage disease (prognostic biomarkers), earlier detection, and monitoring of metastatic disease load (surveillance biomarkers) and treatment planning (predictive biomarkers). This article comprehensively revises evidence supporting the potential clinical utility and/or biological context of BrM biomarkers, highlighting the most recent advances in both pre-clinical and clinical settings.

## 3. Biomarkers for Prognostication and Differential Diagnosis of BrM

Measuring changes in the expression of individual proteins or multi-gene signatures has dominated the landscape of prognostic biomarker research. These studies often share a common goal of being able to predict the risk of BrM at the point of primary cancer diagnosis. For example, Duchnowska identified a 13 gene BrM prediction signature for HER2+ breast cancer [28], and this was further refined to a 3 gene classifier that included *RAD51* (RAD51 homolog), *HDGF* (hepatoma-derived growth factor), and *TPR* (translocated promoter region) genes. Interestingly, multivariate analysis revealed that this 3 gene signature was highly predictive of early BrM in the discovery cohort, but it was not confirmed in the validation cohort. These results indicate that significant differences between patient cohorts may impact the genes expressed that drive the metastatic pattern. There were differences in the linic-pathological characteristics, treatment regimens, and number of patients, which may have resulted in the non-confirmation of the signature. Nonetheless, it identified few crucial genes which might lead to BrM development. Kamer et al. discovered a similarly indicated signature for lung cancer, which additionally predicted metastatic spread with and without brain involvement [29]. The genes involved metastatic spread to the brain from the lungs were mainly oxidative phosphorylation pathway genes, indicating that perhaps in BrM, a more efficient way of utilising glucose which is highly available in this microenvironment is necessary to meet the demands of the rapidly growing metastasis. Prognostic protein biomarkers have also been explored, offering the key practical advantage of rapid integration into current clinical diagnostic practice as most laboratories already have cost-efficient processes for immunohistochemistry (IHC). For example, expression of NDRG1 (N-myc downregulated gene 1) is higher in BrM-initiating breast tumour cells [30], and expression of PLEKHA5 (Pleckstrin homology domain-containing A5) in melanoma is associated with brain-specific metastasis [31]. The detection of markers such as PLEKHA5 and NDRG1 in primary tumour biopsies could provide an early indication of aggressive phenotype, providing they can be fully validated. Practical limitations on prognostic biomarker translation are expertly reviewed elsewhere [32,33].

To be implemented in clinical diagnostic practice, biomarker discoveries require validation in independent sample cohorts, reaching acceptable levels of sensitivity, specificity, and feasibility in a single sample assay context, as well as favourable cost–benefit analysis. Few biomedical research discoveries proceed to this stage, but those that do tend to focus on patient subpopulations where clinical decision making is not optimally served by existing diagnostic frameworks. One such group is BrM patients whose primary tumour type cannot be unequivocally identified by existing differential diagnosis approaches. The frequency of cancers of unknown primary (CUP) may be as high as 16% of all BrM in some institutions [34]. Patients with no history of prior malignancy potentially benefit the most from accurate diagnostic information at this stage, as in theory, their disease is still sensitive to first-line agents. 

Genomic mutation profiling is often used for CUP diagnosis, especially in patients with widespread metastatic disease, providing a BrM tissue sample is available. For example, epidermal growth factor receptor (*EGFR*) mutations and *ALK* rearrangements are usually indicative of a lung cancer origin (most CUP cases), while *BRAF* and *NRAS* mutations are associated with melanoma. *EGFR* mutations are also prognostic after BrM diagnosis [35,36]. Multigene signatures may have a role, providing there is infrastructure for these assays. Zheng et al. developed a 90 gene signature that distinguished between primary and metastatic brain tumours with 99% accuracy, and for BrM, correctly predicted primary tumour histology 89% of the time [37]. 

Cerebrospinal fluid (CSF) is gaining credibility for brain tumour diagnostic applications [38], with some studies reporting that biomarkers might be more abundant in CSF than peripheral fluids [39,40,41,42]. Next-generation sequencing of ctDNA from CSF of 53 patients revealed that >50% of samples harboured somatic alterations specific to brain metastatic disease [43]. Other studies have also demonstrated the diagnostic utility of CSF ctDNA analysis, with mutation profiles generally consistent with the primary tumour type (e.g., *NRAS* and *BRAF* mutations from melanoma patients and *EGFR* mutations for lung BrM patients [42]). These studies highlight the potential of CSF as a biomarker identification medium, but CSF sampling is considered highly invasive. It may be a suitable source of biomarkers only if other circulating biomarkers are uninformative. It may also be possible that CSF provides BrM-specific diagnostic information, but this is yet to be demonstrated in a clinical setting.

## 4. Surveillance Biomarkers

The majority of BrMs are diagnosed at an advanced stage when patients already have neurological symptoms. If there are also other comorbidities from extracranial disease, this poses major challenges for disease management, and the primary goal of treatment in this setting is to stabilise, rather than cure, disease. Current diagnostic tools are unable to account for tumour heterogeneity or track progression without multiple, lengthy imaging appointments. More efficient alternatives are needed to enable regular monitoring and expedite early screening. A non-invasive mode of disease monitoring that is gaining momentum is the regular screening of whole blood or blood products for circulating biomarkers, including circulating tumour cells (CTCs), cell-free DNA (cfDNA), and extracellular vesicles [44,45,46]. These are further discussed below, and Table 1 lists the specific emerging biomarkers that may be relevant for early identification of BrM, or for monitoring disease load after treatment has been initiated.

### 4.1. Circulating Tumour Cells (CTCs)

CTCs are tumour cells shed from a solid mass into the circulation (blood and lymph). They are thought to be the opportunistic ’seeds’ of metastatic progression capable of forming micro-metastatic reservoirs [75,76,77]. Evidence indicates their systemic load correlates roughly with the extent of spread, making CTCs a relevant biomarker for monitoring systemic disease load [78]. In 2018, Hanssen explored the predictive value of CTCs in lung cancer patients with oligometastatic brain disease, reporting a significantly worse prognosis for patients with a higher CTC load [79]. The main challenge translating this knowledge into a clinical practice has been the quantification of CTCs, due to their low overall count in the blood. Various in vitro and in vivo strategies are being tested to overcome this, but it remains a persistent barrier to clinical implementation. 

Currently, there is just one commercially available, FDA-approved platform for CTC counting—CellSearch^®^, manufactured by Veridex. It uses an antibody against epithelial cell adhesion molecule (EpCAM), which is present on carcinoma cells but not on normal blood cells [80]. The commercial platform does not detect EpCAM-negative CTCs [81], but Huebner recently developed a filtration-based system with better sensitivity [82]. It involves filtering CTC subpopulations and then sorting them by flow cytometry, an approach that seemed to have greater prognostic value for metastatic breast cancer patients with overall low CTC load. This filtration system is based on an automated nucleic acid preparation system (VERSANT^®^ kPCR), which can also simultaneously purify DNA, RNA, or proteins from CTCs for further analysis. 

Single-cell CTC profiling has highlighted metastasis-specific alterations that could be targeted to maximise sensitivity [83,84]. For example, in a study of breast BrM patients, Boral et al. identified exclusive elevated Ki67 expression as well as other cell death and immune evasion pathways in BrM-CTCs compared to non-BrM CTCs [50]. The differential expression of these pathways in BrM-specific CTCs highlight potential applications in detection of early stage BrM.

### 4.2. Circulating Cell-Free DNA 

Circulating, cell-free DNA (cfDNA) released from tumour cells may be a powerful biomarker of BrM. Its abundance increases as the integrity of the BBB is progressively compromised in expanding tumours. In contrast to CTCs where sensitivity is often an issue, a major consideration for cfDNA-based surveillance is specificity—distinguishing cfDNA from normal and tumour cells. Circulating tumour DNA (ctDNA; specifically shed from tumour cells) constitutes around 1% of total cfDNA and can be distinguished on the basis of size and genetic profile. ctDNA fragments are shorter than cfDNA from normal cells and harbour somatic mutations [85,86], making ctDNA an ideal diagnostic biomarker. Furthermore, it can be readily detected in plasma or cerebrospinal fluid using droplet digital PCR (ddPCR)—a precise, sensitive, and robust technique that is also relatively user-friendly for the clinical diagnostic setting.

In early stage melanoma, serum lactate dehydrogenase (LDH) is routinely assayed because early intervention based on elevated LDH improves patient outcomes. Using ddPCR to detect *BRAF* or *NRAS* mutations in ctDNA of metastatic melanoma patients, Chang et al. found that mutated ctDNA was elevated in 83% of patients with BrM, whereas LDH was elevated in only 50% of cases. Hence, at least in this study, ctDNA had significantly higher sensitivity than LDH for monitoring disease progression [87]. With respect to clinical translation, a randomized trial with paired CT scans and ctDNA plasma sampling in bevacizumab-treated patients with non-resectable metastatic melanoma is ongoing (NCT02872259). Because of the reduced life expectancy, CNS involvement is an exclusion criterion that limits the study to extracranial disease monitoring. Nonetheless, melanoma BrM patients are still likely to benefit from a ddPCR-based cfDNA test being implemented, even if it is on the basis of extracranial monitoring data.

### 4.3. Extracellular Vesicles

Extracellular vesicles (EVs) are small membrane-bound organelles that are released from cells. They transport different cargoes through the circulation, including proteins, nucleic acids, lipids, miRNAs, and metabolites. Exosomes are small EVs involved in cell–cell communication. As a substrate for disease monitoring, exosomes are becoming more mainstream rather than a niche research field. According to a market report from Grand View Research, exosome research and their clinical use will be worth USD 2.28 billion by 2030, representing an annual growth rate of 18.8% [88]. Several preclinical studies reported a crucial role for exosomes in brain colonisation and the BrM TME [61,72,89,90,91,92]. EVs are isolated via density gradient ultracentrifugation or size-exclusion chromatography. Both are specialised and labour-intensive, being unsuitable for a clinical diagnostic laboratory. Newer technologies that circumvent the current practical limitations will be critical for implementation in the clinic, as will standardized protocols for isolation and analysis.

BrM-derived exosomes that contribute to BBB dysfunction through miRNA-181c may be a substrate for surveillance, as serum miR-181c is elevated in metastatic breast cancer patients with BrM [92]. Similar studies have reported EV or exosome-mediated tumour progression, but their potential for surveillance has not been investigated. For example, EV-derived miRNA-122, involved in metabolic reprogramming [91], exosomal miR-19a released by activated astrocytes in the BrM TME [61], and exosomal CEMIP (cell migration inducing hyaluronidase 1) protein whose uptake by brain endothelial and microglial cells induces pro-inflammatory cytokines and vascular remodelling [72]. 

## 5. Predictive Biomarkers for Treatment Planning

Predicting treatment responses in central nervous system tumours is highly valuable and the beneficial outcomes of this has been widely discussed elsewhere [93,94,95,96]. Overall precision cancer medicine is an evolving but accepted new norm in many parts of the developed world [97,98]. The paradigm is supported by basket trials that established the benefits of treating cancer patients on the basis of their specific mutation profile rather than histology alone [99]. BrM patients can benefit from precision care in centres where this is routine, although neurosurgical tissue is not always available. It is possible that predictive information could be garnered from a liquid biopsy, but the feasibility and accuracy of this route have not yet been demonstrated. A primary tumour biopsy may be the next best option at present, with the caveats that BrM outgrowth potentially not being dependent on targetable alterations identified in the primary, and that BrM-specific or clonal alterations may be missed.

Brastianos et al. performed whole-exome sequencing on 86 BrM and matching primary tumour samples, predominantly from breast, lung, and kidney cancer patients. The divergent genetic profiles of sample pairs illuminated the dynamics of clonal evolution during progression, but also highlighted possibilities to personalise the prescription of molecular-targeted therapy, with BrM samples frequently harbouring alterations that confer sensitivity to PI3K/AKT/mTOR, CDK, and HER2/EGFR inhibitors [100]. Our own genomic analysis of Australian cases concurred, with actionable mutations identified in 86% of BrM analysed [101]. A more recent study by Tyran and colleagues confirmed the benefits of genomic testing on BrM samples rather than primary tumours, where available [102].

Another potential target in BrM is homologous recombination deficiency (HRD). Most often caused by pathogenic mutations in *BRCA1* and *BRCA2*, HRD compromises the repair of double-stranded DNA breaks, exacerbating overall genomic instability and increasing the dependence on poly-ADP-ribose polymerase (PARP)-dependent repair. Exploiting a synthetic lethality between HRD and *BRCA1/2*, PARP inhibitors such as olaparib are now used to treat familial ovarian and breast tumours with this genotype [103,104]. In metastatic breast cancer, HRD was found to be higher in BrM than the primary tumour [105]. Therefore, the use of PARP inhibitors in the BrM setting could be an important ancillary application worth investigating. Other studies revealed elevated HRD and mismatch repair deficiency signatures in BrMs compared to matching primary breast and colorectal cancers [102,106]. 

Human epidermal growth family receptors (HER) have been extensively studied in BrMs by our group and others [101,107,108,109]. HER3 and HER4 activation are elevated in BrM, but not EGFR, suggesting this is a microenvironment-driven feature since neuregulins are abundant in the brain but EGF is not [101,107,108]. Similarly, RNA sequencing of longitudinally collected BrM from breast cancer revealed elevated *RET* and *HER2* signalling. Their inhibition reduced proliferation in patient-derived tissue cultures and significantly slowed the growth of matching patient-derived xenograft (PDX) models [110]. Hence, TME-dependent changes could be exploited by dual targeting of these proteins along with PI3K inhibitors [109]. 

The advent of immunotherapy targeting immunomodulatory proteins such as programmed cell death protein 1 receptor (PD-1) and its ligands has shown promise in many cancers [111,112]. With respect to BrMs, a phase II clinical trial on melanoma BrM patients [113] and phase I on NSCLC BrM patients [114] showed the combined use of ipilimumab and nivolumab resulted in an improved clinical response compared to individual monotherapies. Despite this breakthrough, many patients do not benefit from immunotherapy. Over the last few years, focus has shifted to deciphering predictive biomarkers for the efficacy of immune checkpoint inhibitors (ICIs). For example, with continued advancement of multiplex immunohistochemical technology, as well as high-throughput sequencing, an array of multifactorial predictive markers can be developed [94]. Tumour mutation burden (TMB) test is an adopted guideline by the NCCN for patients with NSCLC receiving immunotherapy. For NSCLC, tumour mutation burden is site-specific, and lung BrMs have the highest TMB. Metastatic sites in lung adenocarcinomas generally had higher TMB with increased PD-L1 positivity [115]. This raises the possibility of investigating ICI treatment in a site-specific manner on the basis of high TMB for NSCLC. However, TMB is not the best predictor of ICI response for many cancers [116]. It should be noted that PD-1/PD-L1 expression and TMB cut-offs may vary across studies, thereby presenting as a challenge to standardize the cut-off criteria for future applications. Furthermore, the feasibility and reproducibility of standardized predictive biomarkers would have to be established to leverage this towards precision immune-oncology for BrMs. 

## 6. Conclusions and Perspectives

The clinical management of BrM patients is a complex challenge that would benefit greatly from more precise diagnostic information at multiple stages of disease progression. Investment should be primarily focused on the development of superior biomarkers for early stage cancer that help prevent BrM in more patients and reduce overall rates of distant relapse, particularly companion diagnostics (predictive markers) that accurately predict the response to individual therapies. There is also great potential for non-invasive monitoring of early cancer patients deemed to be at high risk of BrM (e.g., through regular blood sampling), as this provides an opportunity to intercept newly metastatic patients as early as possible, improving the likelihood of a complete response to second-line therapy and improving patient quality of life. Those with established metastatic disease who are undergoing treatment also stand to benefit from real-time surveillance via blood sampling because it has the potential to be more sensitive and cost-effective than re-staging by PET/MR imaging. There is a very strong rationale for additional research and development on surveillance biomarkers, as well as technology that helps to overcome the practical barriers currently blocking the realisation of this approach in clinical practice.

## Figures and Tables

**Table 1:** Various biomarkers and their sources with applications as predictive and prognostic biomarkers of brain metastasis. The advantages and disadvantages have also been listed [35,47,48,49,50,51,52,53,54,55,56,57,58,59,60,61,62,63,64,65,66,67,68,69,70,71,72,73,74].

Biomarker	Source	Applications	Advantages	Disadvantages
**Circulating** **Tumour Cell**	Blood	- Prognosis [47] - Drug susceptibility [48] - Early diagnosis [49] - Monitoring therapeutic - responses [50]	- Easily collected and less invasive - Cost efficient - Tumour burden estimation - in-vitro and in-vivo assays	- Low concentrations - Not all CTCs generate BrMs - False positives with low purity - Variability in size and density
**Circulating Tumour DNA**	Blood Plasma CSF	- Diagnosis & therapeutics [51,52] - BrM prognosis [53] - BrM driver gene signature [54]	- Non-invasive & cost efficient - Highly specific - Detects intra-tumoral heterogenicity - Treatment resistance prediction	- Hard to distinguish from non-tumour DNA - False negatives & positives - Lack of standardisation
**Circulating** **miRNA**	CSF Plasma Serum Urine Exosomes	- Breast cancer BrM prediction [55] - Treatment response monitoring [56] - BrM detection [57] - BrM formation and targeting [58]	- Highly stable & non- invasive - Early detection & easy to isolate - High specificity for the tissue of origin. - Multimarker model for diagnosis	- Low concentration - Off target effects - Variability in effectiveness - Lack of standardisation
**Extracellular Vesicles**	Tissue Blood CSF Immune cells	- Inhibition of metastasis [59]. - Delivery and breach BBB [60] - Targeting exosomes in BrM [61]	- Moderately Stable - Cell to cell communication - Low immunogenicity - Multiple drug delivery possible	- Less data on mechanism of uptake - Low retrieval concentration - Laborious isolation techniques - Lack of characterisation standards
**RNA**	Tissue Blood	- Target identification [37,62,63,64] - Prediction of BrM progression [65] - Primary tumour classification [66]	- Easy isolation - Widely used & cost effective - Easy to modify and target	- Off target effects - Functional diversity - Requires specific - optimisation - Does not reflect tumour heterogeneity
**DNA**	Tissue Blood CSF	- Epigenetic marker in BrMs [67] - DNA methylation therapeutic targets [68,69] - Predicting mutation pattern [70]	- Accurate gene function exploration - Good Stability - Can monitor therapeutic efficacy - Suitable for pharmacodynamics	- Not highly reproducible - Limited Starting material - Does not reflect tumour heterogeneity - Requires isolation optimisation
**Protein**	Tissue Blood Urine CSF	- Early detection marker [71] - Targeting exosomal protein [72] - Therapeutic targets [73,74]	- Localisation information in tissues - Standardised technique such as IHC - High reproducibility - Provides key pathological data - Insight on disease related pathways	- Difficult to obtain tissue biopsy - Antibody optimisation - Limited starting material - Invasive and expensive
